# Barriers to Health Workers in Iron Deficiency Anemia Prevention among Indonesian Pregnant Women

**DOI:** 10.1155/2020/8597174

**Published:** 2020-12-24

**Authors:** Darmawati Darmawati, Tongku N. Siregar, Hajjul Kamil, Teuku Tahlil

**Affiliations:** ^1^Graduate School of Mathematics and Applied Science, Universitas Syiah Kuala, Aceh 23111, Indonesia; ^2^Department of Maternity Nursing, Faculty of Nursing, Universitas Syiah Kuala, Aceh 23111, Indonesia; ^3^Faculty of Veterinary, Universitas Syiah Kuala, Aceh 23111, Indonesia; ^4^Department of Management Nursing, Faculty of Nursing, Universitas Syiah Kuala, Aceh 23111, Indonesia; ^5^Department of Community Nursing, Faculty of Nursing, Universitas Syiah Kuala, Aceh 23111, Indonesia

## Abstract

**Background:**

Anemia is a global maternal health problem that commonly occurs in developing countries. During pregnancy, a woman will receive antenatal services to check her condition and prevent complications. This study aimed to explore barriers towards achieving eradication of iron deficiency anemia among pregnant women in Aceh Besar District, Indonesia.

**Methods:**

This qualitative study was conducted on 18 health workers who were recruited through a purposive sampling method. Data were collected through in-depth interviews using open-ended questions to gain insight about participants' experiences in managing iron deficiency anemia among pregnant women. Data analysis was conducted by an inductive content analysis method to evaluate, encode, and analyze the interview's result.

**Result:**

Three main themes emerged: (1) facilities, infrastructures, and supplement support; (2) sociocultural factors; and (3) health provider competency deficits and no developing guidelines.

**Conclusion:**

Our findings provide understanding that there are many obstacles and barriers encountered by health workers in iron deficiency anemia prevention management. Thus, the management of anemia must be supported by a skilled health worker and quality facilities. Health workers and pregnant women must work together to achieve optimal management of anemia prevention.

## 1. Introduction

Anemia during pregnancy is a major health problem that often occurs in developing countries, and this situation can cause negative effects on pregnancy [1]. Anemia is a risk factor that contributes to 50% of maternal deaths [2]. The World Health Organization (WHO) defines a pregnant woman is classified into anemia if her hemoglobin (Hb) level is less than 11 gr/dl [3]. Globally, anemia in pregnancy is a severe hematologic disorder affecting 32.4 million pregnant women [4]. According to the WHO, anemia is considered to be a significant health problem if its prevalence in a study population is 5.0% or more. The prevalence of anemia ≥40% in a population is classified as a severe public health problem [5]. Currently, Indonesian prevalence of anemia during pregnancy in 2018 was 48.9%; this number increased from the last 5 years which was 37.1% [6, 7], with the prevalence in Aceh Besar district being 37.1% [8]. This condition is quite worrying, so it needs actions and research studies that can be used to overcome this problem. A study conducted by Woldegebriel et al. found that the maternal age, education, family income, religion, number of family members at home, and number of children were determinant factors that contribute to anemia in pregnancy [9].

Iron deficiency is the commonest nutritional problem causing anemia among pregnant women [10]. Half of all the cases of anemia can be attributed to iron deficiency [11]. Iron deficiency anemia (IDA) during pregnancy is associated with adverse health effects, including low birth weight and preterm birth. Iron deficiency at giving birth is also associated with developmental delays in children [12]. Also, adverse effects felt by the mother include fatigue, decreased body's immune system functions, poor work capacity, increased risk of heart disease, and death [13–15]. A study also showed that anemia during pregnancy contributed to 23% of indirect causes of maternal death in developing countries [13]. Due to the significant influence of anemia during pregnancy on maternal death, this condition must be treated immediately. This is also in line with one of the targets in Sustainable Development Goals (SDGs) which try to reduce maternal mortality until less than 70 per 100,000 live births in 2030 [16].

Pregnant women must prepare properly for their pregnancy and receive adequate antenatal care. Antenatal care is a program that becomes a way for pregnant women to know their condition so that health workers may be able to provide services based on what they need [2]. Previous studies also found that the quality of antenatal care and number of visits can prevent anemia during the prenatal period [17]. It shows that the role of quality antenatal care in preventing anemia cannot be ruled out [18]. During this time, the Indonesian Ministry of Health has been trying to improve the quality of antenatal services provided to pregnant women. Currently, antenatal care standards that apply in Indonesia consist of 11 procedures that must be met by health workers [19]. The procedures are (1) weight measurement, (2) upper arm circumference measurement, (3) blood pressure measurement, (4) fundal height measurement, (5) fetal heart rate measurement, (6) determine fetal presentation, (7) provide tetanus toxoid immunization, (8) provide iron tablet, (9) provide laboratory test, (10) provide referral properly, and (11) provide health education.

So far, the general antenatal services provided have not been able to manage anemia in pregnancy comprehensively. This is evidenced by the prevalence of anemia that tends not to decrease. The reason why this procedure cannot overcome the pregnancy complications is still not clear enough. Previous studies have not examined barriers perceived by health workers in undergoing IDA prevention management among pregnant women. More insight about these barriers needs to be studied more deeply, so it can support policy-making to improve the quality of antenatal care and suits the needs of pregnant women. The purpose of this study was to explore barriers towards achieving eradication of iron deficiency anemia among pregnant women in Aceh Besar District, Indonesia.

## 2. Materials and Methods

We used a qualitative method with in-depth interviews to explore the barriers perceived by health workers regarding IDA prevention management among pregnant women. The data were collected in Public Health Centers (PHCs) in Aceh Besar District, Indonesia. In total, there are 28 PHCs in this area. This study was done at 9 selected PHCs in Aceh Besar district and selected using a simple random sampling method. The participant recruitment process was done using purposive sampling methods. The purpose was to recruit participants using inclusion criteria, which were health workers with a minimum diploma level of education, worked at the selected Public Health Center, and responsible for the Mother and Child Health Department. There were 18 health workers who met these criteria and participated in this study.

In-depth interviews were conducted using open-ended questions. The questions used in this study include the following: (1) what is the incidence of anemia in your work area? (2) What are the PHC programs related to the management of anemia in pregnant women? (3) So far, what are the obstacles faced by health workers in managing and preventing anemia among pregnant women? (4) Do PHC have a special counseling program for pregnant women about anemia that been designed at this PHC? (5) What do you think if anemia counseling is designed separately from other conditions during pregnancy? (6) Have you previously trained to be an anemia counselor? (7) What do you think if there is a health worker who is prepared to do counseling about anemia in pregnant women? (8) Are there any suggestions from you to minimize the obstacles found in managing and preventing anemia in pregnant women? The data collection process started with recruiting participants from the selected PHCs. After informed consent was obtained and the participants agreed to participate in the study, interview began. The interview was held for 45–60 minutes. All information from the interview was recorded with digital voice recorders and noted by hand. Interviews were held in the health center hall to minimize noise. After a number of participants were obtained, data collection was stopped because no more new themes were found, so we concluded the data saturation had been achieved.

The inductive content analysis (ICA) method was used to evaluate, encode, and analyze the results of verbal interviews. The results of interviews and discussions were listened several times before being written into a transcript. After that, the results of the transcript were read several times to get a good contextualized understanding. Furthermore, a list of categories and subthemes was developed based on research objectives to answer the whole research questions [20]. Another investigator independently reviewed and verified these categories and subthemes. This study was approved by the Ethics Committee of the Nursing Faculty, Universitas Syiah Kuala, Aceh, with code number 1130041111218.

## 3. Results

In this study, the majority of the participants was midwife and had experience in providing health services, especially antenatal care for pregnant women (Table 1). The codes were classified into themes, categories, and subcategories. In the process of analyzing qualitative data after categorizing the code and removing the same code, 12 codes were obtained in 10 subcategories, 7 categories, and 3 main themes, which were facilities, infrastructures, and supplement support; sociocultural factors; and health provider competency deficits and no developing guidelines (Table 2).

### 3.1. Facilities, Infrastructures, and Supplement Support

Health workers in this study revealed that they felt the facilities and infrastructure in IDA prevention management were inadequate. They stated that the place being not appropriate and not specific becomes the inhibiting factors for the counseling process, as they said:

*“During this time, counseling is done in the nutrition room. Actually, the room is very narrow and uncomfortable for counseling. Sometimes it also feels hot*, *too*.*”* (P2)

Most of the participants revealed that only general funds were provided for maternal and child health. There were no special funds for the treatment of iron deficiency anemia. These limited funds were barriers for them in providing services to prevent iron deficiency anemia among pregnant women, as they said:

*“The first obstacle may be from the funds…”* (P6)

*“Another obstacle I think is about the funds. There are funds but for the mother and child health overall, there are no special funds for anemia*.*”* (P4)

Some health workers stated that there were no clear calculations for the availability of iron tablets and hemoglobin measuring devices. As a result, there are too many iron tablets that have expired, and one component of the hemoglobin examination tool was not enough, which was the strips.

*“For iron tablets, there are many in our place (in the Public Health Center). But because it supplied too much, so many of them now are out of date*.*”* (P7)

*“Yes, we have the Hb meter tools. But the sticks aren't available. The amount is not enough*.*”* (P11)

### 3.2. Sociocultural Factors

Most of the participants revealed that the behavior of pregnant women and families poses a special obstacle for health workers to make sure they follow the advice stated by health workers. This sociocultural factor was very strongly held by pregnant women and their families. They had incorrect views in consuming iron tablets so that they become not compliance in consuming them, as they said:

“*Most of the behavior is not compliance in consuming iron tablets*.” (P1)

“*I know they think they are given medicine, not supplements, they consider themselves not sick so they do not want to consume drugs…*” (P4)

In addition to the view of iron supplementation, the majority of participants also revealed that pregnant women continue to consume the wrong foods during pregnancy, which were foods that can inhibit iron absorption and trigger anemia, as they said:

*“Many pregnant women drink coffee or tea. Even though she was told not to drink it during pregnancy. But they still drink*.*”* (P10)

Participants in this study also stated that there was a wrong view of society in perceiving a woman's pregnancy. Many pregnant women feel ashamed if their pregnancy is known to other people, and the husband also felt embarrassed to accompany a pregnant wife to do a pregnancy checkup, as they said:

*“When she was in the first trimester of pregnancy, she was hiding from health workers, decided not to do a pregnancy check because they were shy if people found out she was pregnant. So, they did not get iron tablets in the first trimester*.*”* (P2)

*“Pregnant women are rarely accompanied by her husband. Maybe only one or two, but most of what I have seen so far, their husbands do not accompany. It is because they embarrassed because their wives are pregnant*.*”* (P15)

Related to social factors, participants revealed that most of the husbands worked as fishermen. However, the work results are not provided as food sources for his family, but sold and replaced with other foods; they even bought instant noodles, as stated by the participants:

“*The catch (the work result as a fisherman) was immediately sold and, if possible, they would not eat the fish for even one piece. The money earned is used to buy other foods, such as indomie (instant noodles)*.” (P9)

### 3.3. Health Provider Competency Deficits and No Developing Guidelines

The majority of participants in this study revealed that there were no specific guidelines on anemia management for counselors. The counseling that was carried out was general counseling with the guidance of the Mother and Child Health book published by the Indonesian Ministry of Health, as they said:

*“There is a guideline for counseling, only from maternal and child health books. The book is general for all conditions during pregnancy, not specific for anemia.”* (P5)

Participants also revealed that there were no special health workers who became anemia counselors. Counselors who have been conducting counseling so far were nutritionists who had never undergone counseling training, including anemia counseling training, as they stated:

*“The person who did the counseling is nutritionist. They have never been trained about counseling, especially for the counseling about anemia*.*”* (P12)

Some participants said that counseling was not carried out by health workers who examined pregnant women, but by the nutrition department. The counseling process was carried out by nutritionists after other health workers examined pregnant women in the Maternal and Child Health room, as they said:

“*Counseling is usually done by a nutritionist. After getting the conditions of what happens to pregnant women, we collaborate with nutritionists*.” (P13)

## 4. Discussion

In the present study, it was found that there were obstacles that were complained by health workers in managing problems in pregnancy, especially anemia. They realized that anemia was a problem that must be handled. However, health workers feel they did not have adequate facilities and infrastructure, felt inadequate, and did not have complete clinical guidelines to manage anemia in pregnancy. The results of this study were supported by the previous study conducted by Widyawati et al. who found that insufficient facilities, high work load, lack of training opportunities, and learning resources for the health care providers are obstacles found in preventing anemia during pregnancy in the PHC [21].

The inadequacy of facilities and infrastructure that support a service in overcoming a health problem was an aspect that should receive more attention. A literature study conducted by Manyisa and van Aswegen found that inadequate infrastructure and resources were the main factors contributing to the decline of the health service quality [22]. Other research also found that poor funding budgets were a major challenge in providing quality health services [23]. The reality that occurs was very contradictory to government programs that seek to reduce maternal mortality rates through improvements in antenatal care standards that have been carried out so far [19]. Improvement of health services, facilities, and financial support should be done especially in the management of anemia because this situation is one of the global health problems that contributes to increasing maternal mortality.

Besides the ineffectiveness of facilities and infrastructure, cultural beliefs held by pregnant women and their families regarding pregnancy were also an obstacle for health workers in conveying health information. A study proved that cultural beliefs that are believed to be true will affect a pregnant woman's daily life [24]. To make pregnant women and their families follow the advice stated by health workers, it is important for health workers to have effective communication with them and their families. When effective communication has established a trusting relationship, it will be easier for them to convey information by aligning cultural beliefs that are believed by pregnant women. When this trust is not built up, pregnant women will be closed and tend not to respect the information provided. It would be better if the pregnant woman was accompanied by her husband or close family so that the information would be conveyed thoroughly. A husband who accompanies his wife when receiving health services will positively correlate with the woman's own behavior in internalizing health-related advice. It is because as the main decision maker in the household, the husband will play a big role in all aspects including the behavior of seeking health assistance [25]. However, a literature study conducted by Chang et al. revealed that, in building a mutual trust relationship, it will also be influenced by the social status of pregnant women, the environment, the number of health workers, and their cultural beliefs [26].

In addition to barriers from facilities and clients, health workers also pointed out that while undergoing their role as information providers to pregnant women, they never had specific guidelines, especially when providing counseling about anemia. They have also never been trained how to do the right counseling. Previous research also found that the lack of quality health services to pregnant women is influenced by the ineffectiveness of work placement of health workers, lack of training opportunities, and learning resources [21]. Increased knowledge and quality of service by health workers are needed. Training programs must be specifically designed to demonstrate and enhance the knowledge and skills of health workers. Furthermore, it must be reevaluated after the training is done. It is done to ensure that good quality health services can still be provided by health workers, especially related to anemia management, so that this condition can be prevented earlier [27]. The training provided must also be able to run effectively. A study conducted by Naeem suggested that it is necessary to refresh knowledge regularly and periodically so that the knowledge possessed is not forgotten and can be updated according to the needs of pregnant women [28].

Overall, this study found important information about barriers perceived by health workers in managing and preventing iron deficiency anemia among pregnant women. Many barriers perceived by health workers can be used as a supporting reference for policy makers at the Public Health Center to make a comprehensive improvement in the management of anemia, both in terms of health care facilities and the ability of health workers. However, there are some limitations in this study. Additional interviews conducted with health workers will enrich the data. In addition, interviews with policy and decision makers at the Public Health Center will also help to explore this issue.

## 5. Conclusion

The effectiveness of managing and preventing iron deficiency anemia in pregnancy is influenced by factors that actually inhibit its application. So far, health workers have experienced many problems in managing anemia in primary care, such as inadequate facilities and infrastructure, incompetent staff, and inhibiting factors from pregnant women and their families. Based on our research findings, we conclude that health providers who provide antenatal care to pregnant women, especially in the management of iron deficiency anemia, need to be cared for and minimize the obstacles found so that the services provided can be more adequate.

## Figures and Tables

**Table 1:** Characteristics of participants.

Characteristics	*n* = 18	%
*Age*		
<40 years old 40–50 years old >50 years old	4 13 1	22.2 72.2 5.6

*Education*		
Diploma Bachelor of Public Health Master of Public Health Medical degree Bachelor of Dentistry	9 3 2 3 1	50 16.7 11.1 16.7 5.5

*Practical experience*		
<20 years >20 years	12 6	66.7 33.3

**Table 2:** Major findings with code, subcategories, categories, and themes.

Code	Subcategory	Category	Theme
Inadequate counseling room Limited funds for the management of anemia	Inappropriate and unspecified places inhibit the counseling process There are no specific funds available for anemia treatment	Inadequate infrastructure in iron deficiency anemia prevention management	Facilities, infrastructures, and supplement support
Hb tool components are not sufficient	Inadequacy of the Hb tool component		
The available iron tablets have expired	Incorrect calculation of iron tablet supply	Inadequate supplement supply	

Noncompliance for iron consumption Eating the wrong food	The behavior of pregnant women in consuming iron	An incorrect view of iron needs	Sociocultural factors
Embarrassed if the pregnancy is known by others			
Ashamed if accompanying a pregnant wife	Community perception about pregnancy	The wrong culture related to pregnancy	
Exchange iron-containing work results with nonnutritious foods	Food sources of iron are not consumed by the family	Bad behavior towards family	

Anemia counselors are not trained	There are no trained health workers to become anemia counselors	Health workers' knowledge and competence	Health provider competency deficits and no developing guidelines
Incompatibility of staff competence in the management of anemia	Incompatibility of health workers' competencies		
No specific guidelines for anemia counseling	No special guidebook available	Specific guidelines for anemia counselors	

## Data Availability

The data used to support the findings of this study are available from the corresponding author upon request.
